# “Big Data” for breast cancer: where to look and what you will find

**DOI:** 10.1038/npjbcancer.2016.31

**Published:** 2016-11-02

**Authors:** Susan E Clare, Pamela L Shaw

**Affiliations:** 1 Department of Surgery, Feinberg School of Medicine, Northwestern University, Chicago, IL, USA; 2 Galter Health Sciences Library, Feinberg School of Medicine, Northwestern University, Chicago, IL, USA

## Abstract

Accessing the massive amount of breast cancer data that are currently publicly available may seem daunting to the brand new graduate student embarking on his/her first project or even to the seasoned lab leader, who may wish to explore a new avenue of investigation. In this review, we provide an overview of data resources focusing on high-throughput data and on cancer-related data resources. Although not intended as an exhaustive list, the information included in this review will provide a jumping-off point with descriptions of and links to the various data resources of interest. The review is divided into six sections: (1) compendia of data resources; (2) biomolecular repository “Hubs”; (3) a list of cancer-related data resources, which provides information on contents of the resource and whether the resource enables upload and analysis of investigator provided data; (4) a list of seminal publications containing specific breast cancer data, e.g., publications from METBRIC, Sanger, TCGA; (5) a list of journals focused on data science that include cancer-related “Big Data”; and (6) miscellaneous resources.

## Introduction

We are living in the era of “Big Data”, which has facilitated many of the breakthroughs that have been witnessed in “Precision Medicine” and will underpin most, if not all, of the treatment and prevention advances yet to come. These data, however, will be of benefit only if they are able to be accessed. Data are produced in any number of settings: by individual investigators/labs, by charities/philanthropies that host and may curate data, by national projects, e.g., The Cancer Genome Atlas, and by international consortia, e.g., The International Cancer Genome Consortium. Data within any one resource can range from a niche data set to fully integrated data produced by multiple technology platforms and thousands of patient samples.

The genesis of this review was our personal interest in finding where breast cancer data can be found and determining the range of data types available. We acknowledge that what we have chosen to highlight in this review reflects our interests and biases; any two other individuals would almost certainly have made different choices. Nevertheless, this survey can provide a glimpse of what is available and provide a jumping-off point from which an exploration of data resources can begin. We apologize to our colleagues whose databases were not included owing to space limitation. All of these databases are “labors of love”, many sustained by the will of their developers, their belief in the value of the data and a shoestring budget. We applaud these efforts and encourage their continuation. The data resources not only facilitate the evolution of “Precision Medicine” efforts but also enable many hypotheses to be tested *in silico*, saving time and money, and maximizing efficiency. In some cases, the data disabuse us of dearly held assumptions and in other cases open new and unimagined avenues of research.

## Compendia of data resources

### Nucleic acids research database summaries

For 23 years, the journal Nucleic Acids Research (NAR) has published an annual database issue, which provides summaries of new databases of interest to biomedical researchers and also describes annual updates to large biomolecular repository “hubs” such as the National Center for Biotechnology Information (NCBI), Swiss Institute of Bioinformatics, European Bioinformatics Institute and the DNA Data Bank of Japan.

The NAR collects database summary papers published in all database issues in a list on the journal’s website: https://www.oxfordjournals.org/our_journals/nar/database/c/. The journal provides a number of ways to search or browse database summary papers: a category list, an alphabetical list, an expanded category/paper list, and a search interface. The categories of databases include nucleotide sequence, RNA sequence, protein sequence, structure, metabolic, and expression. Of particular use to cancer researchers is the “human genes and disease” category, which has a subcategory dedicated to cancer gene databases.

The NAR is an open access journal, so the database summaries are available to access without a subscription. In addition to the database issue, for the past 13 years NAR has published an annual web server issue, which describes and summarizes web-based software tools for the analysis and visualization of biological data.

### Online bioinformatics resources collection

The online bioinformatics resources collection is an online database of bioinformatics resources maintained by the University of Pittsburgh Health Sciences Library System. It can be accessed at the URL https://www.hsls.pitt.edu/obrc/. The online bioinformatics resources collection searches across the NAR database and web server issues and also contains entries from the former BioMed Central databases collection. It also retrieves PubMed search results for terms entered in the search box.

### PLoS Biol 2016; 14(3): e1002418

We also direct the interested reader to Tables 1 and 2 of Kovalevskaya *et al.*^1^ Table 1 provides a list of repositories where researchers can download or upload genomic data and Table 2 provides a list of downloadable genomic data collections.

## Biomolecular respository “Hubs”

### NCBI entrez

The NCBI is the NIH’s large storehouse of electronic biological information and data. Many researchers are familiar with the nucleotide and protein sequence records stored in GenBank, dbSNP, the PubMed literature database, and investigators worldwide use NCBI’s BLAST portal. A powerful tool for researchers at the NCBI’s website is often overlooked: the NCBI sitewide search, known as Entrez.

Entrez is accessed through the NCBI’s main URL: http://www.ncbi.nlm.nih.gov. Users may enter a gene symbol, disease name, or any phrase into the search box, and the Entrez system will search across NCBI’s databases and tools to retrieve result counts for each of 39 databases at the NCBI site.

Of particular interest to cancer researchers are dbGaP, a database of genotype–phenotype correlation data; data sets available in GEO Datasets; ClinVar, a database of clinically relevant variants.

Entrez global search is an excellent way to begin a survey of the available information on a gene or concept of interest, and the results will often lead users to explore their concept in more ways than can be discovered through a literature search or from GenBank records alone.

For additional information about the NCBI, please consult the annual overview and update in The 2016 database issue of *Nucleic Acids Research*.^2^

To learn more about the other two hubs, The Swiss Institute of Bioinformatics and The European Bioinformatics Institute, the interested reader is directed to their websites www.isb-sib.ch, and http://www.ebi.ac.uk, respectively, and to their annual overviews and updates in the 2016 database issue of *Nucleic Acids Research.*^3,4^

## Cancer-related data resources

Supplementary Table S1 lists cancer-related data resources. This table provides the URL, a brief summary of the content, analysis tools available, references, availability of online tutorial, and when the database was last updated. In addition, we highlight a few resources that stand out because of the amount of information that is integrated within the database.

### CanSAR

The canSAR is an integrated knowledge base that incorporates molecular biology, chemistry, pharmacology, structural biology, networks, gene ontologies and clinical trial information. Although this database will be of great utility to those engaged in drug design, it will also have great appeal to the generalist. The canSAR can be accessed via a number of entry points. One can search on genes and proteins, which will return information on whether the protein is a drug target, whether there are bioactive compounds available, whether there is a structure available, whether it is druggable on structure considerations, by ligand-based assessment and/or by network, whether it is an enzyme, and whether mutation and RNA interference data are available. A search on diseases will list 12 cancers including breast. The search will return the number of total clinical trials as determined by cancer.gov and clinicaltrials.gov, number of clinical trials with and without drugs, the number of cell lines models per COSMIC, the number of compounds tested as determined by ChEMBL, the number of active compounds, the number of compounds in clinical trial, and the number of FDA-approved drugs. The clinical trials can be searched by the drug or the NCT ID. Information regarding each individual compound includes chirality, compliance with the “Rule of Five”, whether the crystal structure was solved in complex, whether it contains a toxicophore, whether it is a prodrug, whether it is a clinical candidate or approved drug, whether the compound contains a “Blackbox Warning”, and method of administration: oral, parenteral, or topical. Cell line search provides copy number variation data (COSMIC), mutations (COSMIC), gene expression data (NCI 60), RNA interference data (Broad), and bioactivity data (ChEMBL). Three-dimensional protein structures are available along with ligand interaction plots, the complex in which the protein was bound for the determination of crystal structure, whether the approved drug was in complex with the protein, and the druggable cavity by both relaxed and strict criteria.

Alternatively, gene/protein data can be accessed by searching on the gene name and, on the results page, clicking on the Synopsis logo. In the left hand margin are links to domains and structures; drugs and clinical candidates; druggability; chemistry; ligand efficiency plot; pathways; family cladogram; interaction network; gene expression data from TCGA, the NCI 60, and array express; gene copy number variation from the Cancer Genome Project; RNA interference; and mutations from COSMIC cell lines and patient samples. In addition to search on gene/protein names, there is the ability to BLAST molecular targets. Tools enable the production of a polyphamacology map, a bioactivity profile for single or multiple compounds, and annotations for a set of protein targets or a set of cell lines; and enable the viewing of a heatmap of gene expression, pathway and gene ontologies, and alignments and superpositions of sequences and structures.

### NCI genomic data commons

Launched by Vice President Biden on 6 June 2016, The NCI Genomic Data Commons (GDC) is a core component of the US National Cancer Moonshot (http://www.cancer.gov/research/key-initiatives/moonshot-cancer-initiative) and the President’s Precision Medicine Initiative.^5^ The data included within the GDC at present are from The Cancer Genome Atlas, Therapeutically Applicable Research to Generate Effective Treatments, the Cancer Genome Characterization Initiative, and the Cancer Cell Line Encyclopedia. Eventually, international data from the International Cancer Genome Consortium and data from NCI Clinical Trials will be added. The GDC will import and standardize genomic and clinical data from legacy NCI programs; harmonize mapping of sequencing data to the genome/transcriptome; implement state-of-the-art methods for identifying mutations, determining copy number, identifying structural variants and quantifying gene expression; provide data for download or computation on co-localized or cloud-based computing clusters; and will be open for upload of new genomic data for comparison with existing data. There are 1,098 breast cases included in the TCGA data set. The data types include annotated somatic mutation, raw simple somatic mutation, gene expression quantification, copy number segment, masked copy number segment, isoform expression quantification, and microRNA (miRNA) expression quantification. Searches can be performed by case facets or file facets, this choice is made on the home page. Case facets include the case identifier, primary site, cancer program, project, disease type, gender, age at diagnosis, alive or dead and days to death, race, and ethnicity. Data facets include data category, data type, experimental strategy, workflow type, data format, platform, and access level. The GDC will maintain an instance of the cBioPortal for Cancer Genomics (Supplementary Table S1) to enable analysis of the GCD data. The Broad Firehose is also being extended to support the analysis of GDC data. For additional information, the interested reader is directed to the GDC website (https://gdc.nci.nih.gov/) and to the documentation and other information available on this site.

### International cancer genomics consortium

Until they are integrated within the GDC, the International Cancer Genome Consortium data are likely to continue to be accessed directly. In addition to breast data from the TCGA, the ISGC includes data from 117 BRCA-UK specimens (triple negative/lobular/other; http://icgc.org/node/827) and 560 BRCA-EU specimens (ER+, HER2−; http://icgc.org/node/819). The data includes copy number somatic mutation, simple somatic mutation, structural somatic mutations, array-based gene expression, sequencing-based gene expression, array-based DNA methylation, sequenced-based miRNA expression, and protein expression. There are three categories for advanced search (https://dcc.icgc.org/search): by donor, by gene, or by mutation. In a mirror of GDC search, donors can be searched by unique identifier, primary site, project, study, gender, tumor stage, vital status (alive/dead), disease status, relapse type, age at diagnosis, type of available data, and experimental strategy. Genes can be searched as a single gene or a set of genes, by type, which includes the multiple types of RNA, pathway, gene ontology, curated gene set (Cancer Gene Census from Sanger) and genomic location. Mutations can be searched by mutation ID, consequence of the mutation to the encoded protein, functional impact of the mutation (high, low, unknown), type of mutation, platform, analysis type, verification status, and chromosomic location. Analysis tools include “Enrichment Analysis”, which identifies statistically significant gene sets (e.g., Reactome Pathway, Gene Ontology) in comparison with the user’s gene set of interest; “Phenotype Comparison”, which enables the comparison of two to three donor sets of interest on the basis of gender, vital status, and age at diagnosis; and “Set Operations”, which produces a Venn diagram of the intersection or union of a molecular anomaly of interest within the various data sets.

## Seminal publications

### METABRIC (Molecular Taxonomy of Breast Cancer International Consortium). (2012) The genomic and transcriptomic architecture of 2,000 breast tumours reveals novel subgroups. Nature 486: 346–352

This is an integrated genomic/transcriptomic analysis of breast cancers for which long-term clinical follow-up data are available.^6^ The discovery set was composed of 997 primary tumors and the data were validated in a second set of 995 tumors. The impact of germline variants (copy number variation and single-nucleotide polymorphisms) and somatic (CNA, copy number aberrations) on gene expression was analyzed. It was also determined whether the aberrations worked in *cis* or in *trans*. The CNAs influencing expression in *cis* explained the greatest variance in gene expression and they were shown to be enriched for driver genes. These “driver” CNAs were used in an unsupervised clustering approach to classify the tumors into 10 Integrative Clusters (IntClust) that have distinct copy number profiles and clinical outcomes. Of note, the clusters straddle more than one intrinsic (PAM50) subtype. This manuscript is also a source of information regarding genomic regions containing deletions.

A more recent follow-on manuscript^7^ examines the most frequently mutated genes in breast cancer. A total of 40 “Mut-driver” genes, comprising both oncogenes and tumor-suppressor genes are identified. These genes are categorized by their association with the ER status of the tumor, by their membership in a pathway or by the function of the gene, by clinical and pathological parameters, and by IntClust. Amplification prevalence, amplification plus mutation, mutation plus LOH, and homozygous deletion of the 40 genes within the 2,433 breast cancer samples is also provided. Patterns of co-mutation and mutual exclusivity are presented including the surprising observation that 15 out of 57 tumors harboring PTEN inactivating mutations also had recurrent PIK3CA mutations. The clonal and subclonal status of the Mut-drivers across the IntClusts and within individual tumors is also given. The association of the mutations in Mut-deriver genes with prognosis is analyzed in a number of ways: By ER status and within IntClust. Intratumoral heterogeneity was analyzed across the IntClusts and relationship between intratumoral heterogeneity and chromosomal instability determined.

### The Cancer Genome Atlas Network. (2012) Comprehensive molecular portraits of human breast tumors. Nature 490(7418): 61–70

Array-based messenger RNA (mRNA) expression, sequencing-based mRNA expression, DNA methylation, single-nucleotide polymorphisms, array-based miRNA expression, and protein/phosphoprotein expression were analyzed in different subsets of specimens from 825 breast cancer patients.^8^ In addition to the identification of significantly mutated genes, the investigators also identified mutually exclusive mutation patterns among various genes. Data are analyzed in a number of different ways including correlations (or the lack thereof) of data from one experimental strategy, e.g., mRNA expression with that from others, e.g., mutations, miRNA expression, etc. Multiplatform analysis is performed based on the RNA intrinsic subtype; other integrated analyses are based on pathways. A summary table provides clinical data, pathway data, copy number variation, DNA mutations, DNA methylation, and protein expression as a function of the mRNA intrinsic subtypes.

### The Cancer Genome Atlas Network. (2014) Multiplatform Analysis of 12 Cancer Types Reveals Molecular Classification within and across Tissues of Origin. Cell 158(4): 929–944

Six different “omics” platforms: Whole-exome DNA sequence (Illumina HiSeq and GAII, San Diego, CA, USA), DNA copy number variation (Affymetrix 6.0 microarrays, Santa Clara, CA, USA), DNA methylation (Illumina 450,000-feature microarrays), genome-wide mRNA levels (Illumina mRNA-seq), miRNA levels (Illumina microRNA-seq), and protein levels for 131 proteins and/or phosphorylated proteins (Reverse Phase Protein Arrays; RPPA) were used.^9^ Twelve different histologic malignancies comprising 3,527 cases had been assayed by at least four of the technology platforms. An integrated subtype classification was developed based on DNA copy number, DNA methylation, mRNA expression, microRNA expression, protein expression, and excluding somatic mutations. Thirteen subtypes were identified, of which two were excluded from additional analysis owing to small numbers. Five of the remaining subtypes display a near one-to-one relationship to the tissue of origin. Breast cancers are divided into two distinct groups: C3-BRCA/Luminal contains ER+ and HER2-positive tumors; C4-BRCA/Basal contains basal-like tumors. Of note, the tissue of origin is the determinative signal in almost all other 12 cancer types, however, breast basal-like cancers are as different from luminal/ER+ breast cancers as they are from cancers of the other tissues. The major genomic determinants, i.e., somatic mutations and copy number changes, were determined for each subtype. The degree of genomic instability was shown to be a major determinant of subtype with C4-BRCA/basal displaying marked instability. The subtypes were also shown to be associated with arm-level copy number changes; 4q and 5q loss were identified in breast basal-like cancer. The expression-based determinants of the integrated subtypes were identified using gene programs developed by the investigators or PARADIGM. Molecular features common to basal-like breast cancer, squamous cell lung cancer and serous ovarian cancer are provided. These features include *TP53* mutation, copy number changes, gene expression, and pathway activation. Differences among these three subtypes with regard to isoforms of TP63 and/or TP73 are also presented.

The data sets and results are available on Synapse (https://www.synapse.org/#!Synapse:syn300013/wiki/70804) to support integrative bioinformatics analysis. The results are also available through several portals including the UCSC Genome Browser, Gitools (http://www.gitools.org/datasets/pancancer12), and MD Anderson’s Next-Generation Heatmaps (http://bioinformatics.mdanderson.org/main/TCGA/NGCHM).

### The NCI Clinical Proteomic Tumor Analysis Consortium and TCGA. (2016) Proteogenomics connects somatic mutations to signaling in breast cancer. Nature 534: 55–62

The paper reports an integrated analysis that adds protein and phosphoprotein abundance data to what is already known about mutation status, CNA, and mRNA abundances.^10^ A total of 105 breast tumors previously characterized by TCGA of which 77 passed quality control are examined in this study. High-resolution tandem mass spectrometry and iTRAQ (isobaric tags for relative and absolute quantification)-based quantification is utilized. A number of single amino acid variants, frameshifts, and splice junctions, including splice isoforms that had been detected as only single transcript reads by RNA-seq are reported. However, the number of genomic and transcriptomic variants that were confirmed as peptides by MS/MS is low consequent to limited coverage at the single amino acid level by the technology as it currently exists. The type of mutation, CNA, RNA-seq abundance, protein abundance by MS/MS and RPPA, and phosphoprotein abundance for TP53, PIK3CA, GATA3, ER, PR and HER2 are presented within the mRNA intrinsic subtypes. RNA-Seq and MS/MS protein expression levels are compared; a number of proteins are identified to have lower protein expression than mRNA levels suggesting post-transcriptional regulation of the abundance of these proteins. The consequences of CNA on mRNA, protein and phosphoprotein abundance in both *cis* and *trans* are examined. There is significant positive (*cis*) correlation for approximately one in three CNA–protein pairs and one in five CNA–phosphoprotein pairs. The significance of the protein data is underscored by the observation that cancer-related genes more frequently appear in the subsets of genes that correlate both on the CNA–mRNA and CNA–protein level than on the mRNA level only. The CNA regions with *trans*-association on the protein level are presented and noted to occur infrequently. The identified proteins are potential regulatory candidates. Using 50 of the PAM50 genes or the 35 genes observed in the proteome, mRNA intrinsic subtypes are demonstrated to be largely recapitulated at the protein level. However, unsupervised clustering of the proteome identified three clusters: basal-enriched, luminal-enriched, and stromal-enriched; HER2-enriched tumors were observed to be distributed across these three clusters. The stroma-enriched subtype is a mix of all mRNA intrinsic subtypes. The genes enriched in the luminal- and basal-enriched protein subtypes are provided. Using phosphorylation status as a proxy for activity, the phosphoproteome was used to develop a signaling a pathway-based classification. Four subtypes are identified: subgroups 2 and 3, for the most part, recapitulate the stromal- and luminal-enriched proteomic subgroups, respectively. Subgroup 4 is an admixture of the basal-enriched proteomic subgroup with some of the luminal-enriched subtype tumors. Subgroup one is completely novel, it is not observed at the mRNA or protein level. Using phosphosite data, activated *PIK3CA* and *p53* mutation signatures are developed. Proteogenomic circos-like plots, referred to as pircos plots, are used to map CNA, RNA, protein, and phosphoprotein data to the genome. This enabled the identification of kinases with significantly increased expression that may be candidate therapeutic targets.

## Journals that publish data

Many journals include data as Supplementary Materials, and there are some relatively recent journals that are dedicated to publishing data or data descriptions exclusively. We have included a list of some journals that are relevant for cancer research. A more comprehensive and somewhat current list of data journals can be found in a manuscript published by Candela in 2015.^11^ Data-inclusive journals are appearing frequently in the publishing landscape, so this list is a sampling of what is available.

Our list is ordered alphabetically, and some descriptions are provided. Journals that publish data or data descriptions exclusively are often not listed in impact factor databases, so they cannot be evaluated by such metrics. The authors are encouraged to investigate individual journals before making the decision to publish data in them. We also have included a category for data repositories. Each of these resources is searchable, and we include identification of those journals that are open access.

### BioMed Central journals

BioMed Central (BMC) is an open access publisher with a catalog of science, technology, and medicine titles. BMC journals are peer reviewed. BMC publishes data sets with manuscripts whenever possible and the publisher has an Open Data policy described at http://www.biomedcentral.com/about/policies/open-data. Many BMC titles publish manuscripts on cancer, including (but not limited to) *Applied Cancer Research, BMC Biochemistry, BMC Bioinformatics, BMC Cancer, BMC Genetics, BMC Genomics, BMC Immunology, BMC Women’s Health, Breast Cancer Research, Cell & Bioscience, Epigenetics & Chromatin, Genome Biology, Genome Medicine, Human Genomics, Molecular Cancer, Molecular Cytogenetics, Stem Cell Research & Therapy*.

Special note: BMC publishes *GigaScience*, an open access, open data journal that links manuscripts to data, software tools, and workflows from all areas of “big data” science. *GigaScience* links to GigaDB, a database holding associated data, and GigaGalaxy,^12^ a Galaxy-based data analysis platform holding workflows and computational methods.

### Genomics Data

Published by Elsevier, *Genomics Data* is a peer-reviewed open access journal that publishes standardized reports of results of microarray and sequencing studies in all types of organisms, along with methods, data QC metrics, data analysis process and algorithms, biological interpretation, and conclusions.

### F1000Research

*F1000Research* is an online open access publishing platform that provides immediate publication of manuscripts, posters, and slides. *F1000Research* uses “transparent refereeing of articles” by assigning referees when manuscripts are submitted, while manuscripts are available immediately for viewing. The data associated with manuscripts are made available on the site.

### PLOS journals

The Public Library of Science (PLOS) publishes a number of open access journals. All PLOS journals are peer reviewed. PLOS has a data access policy that requires authors who publish manuscripts in their journals to make their data publicly available. There are some exceptions to this policy, but this data access policy ensures that data accompanying more recent PLOS manuscripts will be available for use. Nearly every PLOS title publishes content on cancer, and PLOS also has a feature called PLOS Collections at their site. Collections gather publications across all titles that fit broad subject categories such as Medicine and Health Sciences.

### Scientific Data

Nature Publishing Group publishes *Scientific Data*, an online-only journal dedicated to publishing data descriptions exclusively. Nature calls articles in *Scientific Data* “Data Descriptors”. Data Descriptors are peer-reviewed, scientific publications that describe experimental or observational data sets. The articles are descriptions only, but authors can deposit their data in a suitable repository and the data descriptor will provide a link to the data. By publishing detailed descriptions of data sets, *Scientific Data* facilitates searching, linking, and reuse of data.

### SpringerPlus

*SpringerPlu*s is a peer-reviewed publication that uses a rapid editorial process. Many types of articles are included in *SpringerPlus*, including types called “Data Note” and “Database Article”. Data Notes are descriptions of data sets and require that the data sets be readily accessible. Database Articles describe novel databases.

### Data repositories

Data sharing policies are increasingly common among scientific publishers, as they strive to provide readers with materials necessary for replication of experimental findings described in their journals’ published manuscripts. For example, the Nature Publishing Group has a comprehensive data policy, which describes requirements for sharing data, materials, computer code, and protocols (http://www.nature.com/authors/policies/availability.html#data). The availability of discipline-specific and generalist data repositories is essential for authors to deposit data supporting their manuscripts to comply with journal requirements and many federal funding agency requirements for data sharing. Many data repositories are available for investigators to deposit or download data. Some of the most-recognized multidisciplinary repositories are figshare (https://figshare.com) and Dryad (http://www.datadryad.org/), but many subject or data type-specific repositories exist, such as GEO Datasets from the NCBI (http://www.ncbi.nlm.nih.gov/geo/), which also provides tools for light data analysis integrated in the site. Data repositories are searchable and data can be downloaded for reuse. An excellent way to find data repositories is to use the Registry of Research Data Repositories (re3data.org), which lists over 1,500 data repositories. Re3Data is searchable or browsable by subject or data type. In addition to these general resources, many journals provide authors with lists of recommended repositories. For example, the SpringerNature combined publishing group supplies recommendations at http://www.springernature.com/gp/group/data-policy/repositories.

## Miscellaneous

Social media is another potential source of information about breast cancer data. LinkedIn hosts a number of groups, for example, Genomics: Next-generation DNA Sequencing (NGS) and Microarray. Twitter is another route to information. The LinkedIn groups tend to be focused on the biotechnology industry and tweets can be redundant, so it may be expeditious to use a social media aggregation site, such as Hootsuite, to display this information on a dashboard where you can take a quick look to see whether there is a new data resource or publication of interest.

## References

[bib1] Kovalevskaya N. V. et al. DNAdigest and repositive: connecting the World of Genomic Data. PLoS Biol. 14, e1002418 (2016). 27011302 10.1371/journal.pbio.1002418 PMC4807091

[bib2] Coordinators NR. Database resources of the National Center for Biotechnology Information. Nucleic Acids Res. 44(D1), D7–19 (2016). 26615191 10.1093/nar/gkv1290 PMC4702911

[bib3] Cook, C. E. et al. The European Bioinformatics Institute in 2016: data growth and integration. Nucleic Acids Res. 44(D1), D20–D26 (2016). 26673705 10.1093/nar/gkv1352 PMC4702932

[bib4] Members SIBSIoB. The SIB Swiss Institute of Bioinformatics' resources: focus on curated databases. Nucleic Acids Res. 44(D1), D27–D37 (2016). 26615188 10.1093/nar/gkv1310 PMC4702916

[bib5] Collins, F. S. & Varmus, H. A new initiative on precision medicine. N. Engl. J. Med. 372, 793–795 (2015). 25635347 10.1056/NEJMp1500523 PMC5101938

[bib6] Curtis C. et al. The genomic and transcriptomic architecture of 2,000 breast tumours reveals novel subgroups. Nature 486, 346–352 (2012). 22522925 10.1038/nature10983 PMC3440846

[bib7] Pereira B. et al. The somatic mutation profiles of 2,433 breast cancers refines their genomic and transcriptomic landscapes. Nat. Commun. 7, 11479 (2016). 27161491 10.1038/ncomms11479 PMC4866047

[bib8] Cancer Genome Atlas Network. Comprehensive molecular portraits of human breast tumours. Nature 490, 61–70 (2012). 23000897 10.1038/nature11412 PMC3465532

[bib9] Hoadley K. A. et al. Multiplatform analysis of 12 cancer types reveals molecular classification within and across tissues of origin. Cell 158, 929–944 (2014). 25109877 10.1016/j.cell.2014.06.049 PMC4152462

[bib10] Mertins P. et al. Proteogenomics connects somatic mutations to signalling in breast cancer. Nature 534, 55–62 (2016). 27251275 10.1038/nature18003 PMC5102256

[bib11] Candela L., Castelli D., Manghi P., Tani A. Data journals: a survey. J. Assoc. Inf. Sci. Technol. 66, 1747–1762 (2015).

[bib12] Afgan E. et al. The Galaxy platform for accessible, reproducible and collaborative biomedical analyses: 2016 update. Nucleic Acids Res. 44, W3–W10 (2016). 27137889 10.1093/nar/gkw343 PMC4987906

