# Supplementary material for: “Big Data” for breast cancer: where to look and what you will find
Source: NPJ Breast Cancer. 2016 Nov 2;2:16031–. doi: 10.1038/npjbcancer.2016.31 (PMC5289822; doi:10.1038/npjbcancer.2016.31)
Supplement: Supplementary Table S1 [file npjbcancer201631-s1.doc]

Table S1 Cancer-related Data Resources

| ArrayMap | |
| --- | --- |
| URL | [http://www.arraymap.org](http://www.arraymap.org/) |
| What you get | copy number data from 8594 breast cancer samples (database includes other tissue types) |
| What you can do | Search samples, search publications, gene CNV frequencies, process user provided data sets |
| Literature & tutorials | PLoS One. (2012) 7(5), e36944. doi:10.1371/journal.pone.0036944  Nucl. Acids Res. (28 January 2015) 43 (D1): D825-D830. doi: 10.1093/nar/gku1123 |
| Update | Approximately biannually |
|  | |
| BCCTBbp:the Breast Cancer Campaign Tissue Bank bioinformatics portal  Now called the BCNTBbp: Breast Cancer Now Tissue Bank bioinformatics portal | |
| URL | <http://bioinformatics.breastcancertissuebank.org/> |
| What you get | Data from genomics, methylomics, transcriptomics, proteomics and microRNA experiments mined from literature, external and internal (Breast Cancer Now Tissue Bank) sources |
| What you can do | Search for data and access via external links. Perform analysis on pathways and variation. (The bioinformatics portal was undergoing migration at the time of this writing, so a more complete description could not be written.) |
| Literature & tutorials | Nucl. Acids Res. (28 January 2015) 43 (D1): D831-D836. doi: 10.1093/nar/gku984 |
|  | |
| BreCAN-Database of Breakpoint profiles of Cancer Genomes | |
| URL | <http://14.139.32.56/> |
| What you get | Somatic DNA breakpoints; 15 (15.2%) breast cancers |
| What you can do | Visualize breakpoints, upload breakpoint profiles to compare with breakpoint hotspots and profiles in database |
| Literature & tutorials | Nucl. Acids Res. (04 January 2016) 44 (D1): D952-D958. doi: 10.1093/nar/gkv1264;  “Help” link available on home page |
| Updated | October 2015 |
|  | |
| Cancer RNA-Seq Nexus | |
| URL | <http://syslab4.nchu.edu.tw/> |
| What you get | Differential expression of coding and lncRNA of various combinations. Data derived from cell lines, tumors and adjacent normal deposited in TCGA and 6 GEO submissions. Total of 1537 breast specimens. mRNA-lncRNA coexpression network available for each pair. |
| What you can do | Download differentially expressed transcripts of the subset pairs |
| Literature & tutorials | Nucl. Acids Res. (04 January 2016) 44 (D1): D944-D951. doi: 10.1093/nar/gkv1282;  Tutorial available on website |
|  | |
| Cancer3D | |
| URL | [http://www.cancer3d.org](http://www.cancer3d.org/) |
| What you get | Cancer mutations mapped onto ribbon diagrams of proteins, mutated AA colored according to their mutation frequency in TCGA; activity of selected drug as a function of location on the protein |
| What you can do | Search on gene or drug; link to protein interaction partners |
| Literature & tutorials | Bioinformatics. (01 November 2014) 30(21):3109-14  Nucl. Acids Res. (28 January 2015) 43 (D1): D968-D973. doi: 10.1093/nar/gku1140  PLoS Comput Biol. (08 January 2015) 11(1):e1004024  PLoS Comput Biol. (20 October 2015) 11(10):e1004518  Tutorial available on website |
| Updated | at release in 2014 |
|  | |
| Cancer PPD | |
| URL | <http://crdd.osdd.net/raghava/cancerppd/> |
| What you get | Lists of proteins and peptides that have demonstrated anti-cancer activities. Searches include protein, peptide, tissue and cell line. |
| What you can do | Blast, align, map |
| Literature & tutorials | Nucl. Acids Res. (28 January 2015) 43 (D1): D837-D843. doi: 10.1093/nar/gku892  Tutorial available on website |
| Updated | October 2014 |
|  | |
| CanGEM | |
| URL | <http://www.cangem.org/> |
| What you get | Gene copy number changes |
| What you can do | Free text search or search on clinical attributes of the specimens of interest, includes stage. Searches can also be performed on the copy number status of a gene. |
| Literature & tutorials | Nucl. Acids Res. (2008) 36 (suppl 1): D830-D835. doi: 10.1093/nar/gkm802  Tutorial available on website |
| Updated | not current |
|  | |
| CaSNP | |
| URL | <http://cistrome.dfci.harvard.edu/CaSNP/index/> |
| What you get | Copy number alteration based on data from Affymetrix SNP arrays (10K to 6.0) available in GEO . Additional data from TCGA, a few individual publications and GlaxoSmithKlein cancer cell lines. |
| What you can do | Search on genomic position, refSeq ID, coordinate range or miRNA ID, cancer type and specific GEO study(ies) |
| Literature & tutorials | Nucl. Acids Res. (2011) 39 (suppl 1): D968-D974. doi: 10.1093/nar/gkq997  Tutorial available on website |
| Update | Last updated in 2011 |
|  | |
| cBioPortal | |
| URL | <http://www.cbioportal.org/public-portal/> |
| What you get | Breast genomic data sets from TCGA, Sanger, Broad and British Columbia; cell line from Cancer Cell Line Encyclopedia and NCI-60 |
| What you can do | Search by mutations, CNA, mRNA and protein expression as available. Identify co-expressed genes and mutation; as well as copy-number, mRNA and protein enrichments. Plot mutation, survival and networks. Generate oncoprints and lollipop plots from investigator data. |
| Literature & tutorials | Sci Signal. (02 April 2013) 6(269):pl1.  Cancer Discovery. (2012) May 1; 2: 401  No abstract in Nucleic Acids Research Database Issue, but this database is NAR Molecular Biology Database Collection entry number 1757  Tutorial available on website |
| Updated | frequently |
|  | |
| CellLineNavigator | |
| URL | http://medicalgenomics.org/celllinenavigator/data |
| What you get | Array-based expression data (Affymetrix HG-U133 Plus2 GeneChip) from ArrayExpress experiment E-MTAB-37 |
| What you can do | Search by gene for relative expression across cell lines from 28 tissue types (19 breast cancer cell lines). Search by combining gene, KEGG Pathway, GO, cell line, organism part and/or disease state (although breast cancer not included among disease states). Gene lists generated in CellLineNavigator may automatically be transferred to the DAVID analysis tool (Bioinformatics 2012;28:1805-1806). |
| Literature & tutorials | Nucl. Acids Res. (2013) 41: D942-D948. doi: 10.1093/nar/gks1012  Basic users’ information available on website |
| Updated | Static dataset |
|  | |
| COLT-Cancer | |
| URL | <http://dpsc.ccbr.utoronto.ca/cancer/index.html> |
| What you get | Genes essential for cancer cell proliferation and survival identified by shRNA screen of 29 breast cancer cell lines. [Data from colon, pancreas and ovarian cell lines also available] |
| What you can do | Search by gene, cell line or essential genes across all cell lines or within a tumor type |
| Literature & tutorials | Nucl. Acids Res. (2012) 40 (D1): D957-D963. doi: 10.1093/nar/gkr959  Documentation available on website |
| Updated | Copyright through 2014 |
|  | |
| COSMIC: Catalog of Somatic Mutations in Cancer | |
| URL | <http://cancer.sanger.ac.uk/cancergenome/projects/cosmic/> |
| What you get | Annotations of mutations in cancer curated from biomedical literature, as well as noncoding mutations, gene fusions, genome rearrangements, abnormal copy number segments and abnormal expression variants annotated to the human genome and correlated across disease types |
| What you can do | Search by gene or keyword. Retrieve results by gene, sample, mutations and literature (PubMed). Full datasets are available for download via SFTP site. Tools include a cancer browser, genome browser, CONAN (copy number analysis tool), Beacon (a tool to search for a mutation at any position in the human genome). |
| Literature & tutorials | Nucl. Acids Res. (28 January 2015) 43 (D1): D805-D811. doi: 10.1093/nar/gku1075  Documentation and tutorials available on website |
| Updated | Updated every 3 months |
|  | |
| Database of Germline p53 Mutations | |
| URL | <http://stary.lf2.cuni.cz/projects/germline_mut_p53.htm> |
| What you get | p53 mutation (type of the mutation, exon and codon affected by the mutation, nucleotide and amino acid change), each family (family history of cancer, diagnosis of LFS), each affected individual (sex, generation, p53 status, from which parent the mutation was inherited) and each tumor (type, age of onset, p53 status (loss of heterozygosity and immunostaining)). Each entry contains the original reference(s). |
| What you can do | View and search Excel spreadsheet |
| Literature & tutorials | Nucl. Acids Res. (1998) 26 (1): 214-215. doi: 10.1093/nar/26.1.214  Documentation available on website |
| Updated | Every 4 months |
|  | |
| intOGen (Integrative Onco Genomics) | |
| URL | <http://www.intogen.org/search> |
| What you get | Mutational cancer driver genes currently numbering 459 |
| What you can do | Search gene to determine if gene is driver and, if so, in which cancer types. Output includes mode of action, mutation frequency and mutation distribution (lollipop diagram). Search by cancer: Breast cancer 184 driver genes identified to date. Analyze list of somatic mutations for a cohort of tumors to identify driver mutations, genes and pathways. Analyze list of somatic mutations from a single tumor to obtain rank ordered list based on implication in ca development |
| Literature & tutorials | Cancer Cell (09 March 2015) 27: 382-396  Nature Methods (15 September 2013) 10: 1081–1083. doi:10.1038/nmeth.2642  No abstract in Nucleic Acids Research Database Issue, but this database is NAR Molecular Biology Database Collection entry number 1909 |
| Updated | Last release number: 2014.12 |
|  | |
| Lnc2Cancer | |
| URL | <http://www.bio-bigdata.com/lnc2cancer/home.jsp> |
| What you get | lncRNAs associated to 93 cancers, method of experimental association, expression pattern, links to Pubmed, sequence, lncrnadb, LncRNA2Function, Co-LncRNA among others |
| What you can do | Search by lncRNA, cancer; download Excel or text file of all experimentally supported LncRNA-cancer association data in database |
| Literature & tutorials | Nucl. Acids Res. (04 January 2016) 44 (D1): D980-D985. doi: 10.1093/nar/gkv1094  Documentation available on website |
| Updated | quarterly |
|  |  |
| MethHC | |
| URL | <http://methhc.mbc.nctu.edu.tw/php/index.php> |
| What you get | DNA methylation, gene expression, microRNA methylation, microRNA expression, and the correlation of methylation and gene expression. Data derived from TCGA |
| What you can do | Search by specific cancer (18), gene, genes in KEGG pathway |
| Literature & tutorials | Nucl. Acids Res. (28 January 2015) 43 (D1): D856-D861. doi: 10.1093/nar/gku1151  Documentation available on website |
| Updated | Last update 2014 |
|  | |
| Mutations, Oncogenes, Knowledge & Cancer (MOKCa) formerly Mutations of Kinases in Cancer | |
| URL | <http://strubiol.icr.ac.uk/extra/mokca/> |
| What you get | Structural and functional annotations of mutations of proteins implicated in cancer. When possible, the database makes predictions on the phenotypic consequences of the mutations. |
| What you can do | Search by COSMIC or Uniprot gene name or accession number. Browse by several categories: whole genome, DNA damage response (DDR), protein kinases, Cancer Gene Census (CGC) molecularly dominant oncogenes, CGC tumor suppressors (molecularly recessive) and drug targets. Retrieve the location of the mutation in the protein sequence and amino acid variant, type of mutation, frequency, domain, modification of residues within 3 residues of the mutation, any PDB structure mapping (if available) and tissue specificity. |
| Literature & tutorials | Nucl. Acids Res. (2009) 37 (suppl 1): D824-D831. doi: 10.1093/nar/gkn832  No help or tutorials available |
| Updated | Last modified Dec. 1, 2015 |
|  | |
| Mouse Tumor Biology | |
| URL | [http://tumor.informatics.jax.org](http://tumor.informatics.jax.org/) |
| What you get | Information on tumors in mice as a model system of hereditary cancer, including endogenous spontaneous tumors and induced tumors, genetic factors associated with susceptibility, and tumor pathology reports. |
| What you can do | Use the Quick Organ/Tissue Search to search by tissue, or use the Search Forms on the left to search by tumor, strain, pathology images, genetics or other criteria. Retrieve tumor name, organ affected, treatment type (if any), mouse strain name, tumor frequency, metastasis sites, images (if any) and a tumor summary. |
| Literature & tutorials | Exp Mol Pathol. (2015) Dec;99(3):533-6.  “Help” link on home page, left navigation menu |
| Updated | Current and actively updated |
|  | |
| MutationAligner | |
| URL | <http://www.mutationaligner.org/> |
| What you get | Mutation “hotspots” identified in protein domains from over 5000 patients and across 22 cancer types. Hotspots are identified from results of multiple sequence alignment of domains that contain missense mutations between analogous residues. |
| What you can do | Search by protein domain, gene or cancer type. View results in a graphical format displaying mutations by residue across the gene for all known mutations or by cancer type. A multiple sequence alignment of similar domains across other genes is also displayed. A full list of mutated positions shown in the alignment is displayed in table format. Download data that underlies all positions. |
| Literature & tutorials | Nucl. Acids Res. (04 January 2016) 44 (D1): D986-D991. doi: 10.1093/nar/gkv1132  Cell Systems (2015) 1(3), 197–209  Link to “FAQ” on home page |
| Updated | Current and actively updated |
|  | |
| Network of Cancer Genes | |
| URL | <http://ncg.kcl.ac.uk/index.php> |
| What you get | Duplicated loci, evolutionary appearance, expression and network properties of protein-coding cancer genes for multiple cancer types |
| What you can do | Search single or multiple cancer genes by symbol, NCBI or Ensembl identifiers; search miRNA-cancer gene interactions with a target gene or miRNA identifier; search for cancer genes in a genomic region. Retrieve information on mutations, orthology, gene expression in normal tissues and cancer cell lines, protein function, duplicated loci, network interactions or miRNA interactions (both viewable in Cytoscape). Browse lists of cancer genes, screenings and possible false positives. Download full list of protein-coding cancer genes. |
| Literature & tutorials | Nucl. Acids Res. (2016) 44 (D1): D992-D999. doi:10.1093/nar/gkv1123  Link to “Help” on home page |
| Updated | Current and actively updated |
|  | |
| SNP500Cancer | |
| URL | <http://snp500cancer.nci.nih.gov/> |
| What you get | 102 resequenced samples to find known or newly discovered single nucleotide polymorphisms (SNPs) which are of immediate importance to molecular epidemiology studies in cancer |
| What you can do | Search by assay ID or Hugo gene symbol, retrieve lists of SNPs on gene, chromosome and start-stop coordinates; click on CGF ID to view annotated sequence and allelic frequency in populations |
| Literature & tutorials | Nucl. Acids Res. (2006 )34 (suppl 1): D617-D621. doi: 10.1093/nar/gkj151  “Help” link on home page |
| Updated | Last dbSNP build used: 130; human genome 36.3 (not current) |
|  | |
| Stem Cell Commons | |
| URL | <http://stemcellcommons.org/> |
| What you get | Open source environment that brings together stem cell datasets, online tools and codes with experiments and their results. |
| What you can do | Browse by organism, disease, cell type or assays; search by keyword; analyze and perform functional pathway analysis in the Refinery platform (still in private beta); visualize NGS data; download code for development and use. Researchers can register as members of the commons to upload their own data to add to the commons. |
| Literature & tutorials | AMIA Jt Summits Transl Sci Proc. 2013 Mar 18;2013:70. eCollection 2013  Help links on home page under development |
| Updated | Current and actively updated |
|  | |
| Stem Cell Discovery Engine | |
| URL | <http://discovery.hsci.harvard.edu/> |
| What you get | Much of the data is being migrated to Stem Cell Commons. See entry for that database. |
| What you can do | At SCDE, users can still use the custom SCDE Galaxy instance with sample data and special tools created by SCDE for pathway fingerprints and gene list comparison. |
| Literature & tutorials | Nucl. Acids Res. (2012) 40 (D1): D984-D991. doi: 10.1093/nar/gkr1051  Link to screencast tutorials on home page |
| Updated | Last modified 05/13/2014 |
|  | |
| SynLethDB | |
| URL | <http://histone.sce.ntu.edu.sg/SynLethDB/> |
| What you get | Information on synthetic lethality gene pairs for 5 species (human, mouse, fruit fly, worm and yeast) collected from literature, experimental assay data and computational prediction |
| What you can do | Enter gene symbol or Entrez gene ID or upload a list of identifiers. Retrieve networks of SL pairs, view interactions and evidence for each pair, retrieve gene set enrichment analysis results based on enrichment results for SL pairs for a given gene, download SL/SDL pair interaction data, launch statistical analysis of drug interactions from SL pair data in tables |
| Literature & tutorials | Nucl. Acids Res. (04 January 2016) 44 (D1): D1011-D1017. doi: 10.1093/nar/gkv1108  Manual available from link on home page |
| Updated | Last modified 10/02/2015 |
|  | |
| TCGA Splice Seq | |
| URL | <http://projects.insilico.us.com/TCGASpliceSeq/> |
| What you get | Cross-tumor and tumor-normal alterations in mRNA splicing patterns of The Cancer Genome Atlas project (TCGA) RNASeq data |
| What you can do | Select from among 33 TCGA tumor types, view splice events for a single gene or for tumor/tumor differences or tumor/normal differences, view splice events in a gene graph or in UniProt sequence display, download PSI data for user-selected splice events by tumor types or for specific genes |
| Literature & tutorials | Nucl. Acids Res. (04 January 2016) 44 (D1): D1018-D1022. doi: 10.1093/nar/gkv1288  Tutorial on site under “FAQ” |
| Updated | Last modified 09/10/2015 |
|  | |
| Tumor Associated Gene database | |
| URL | <http://www.binfo.ncku.edu.tw/TAG/GeneDoc.php> |
| What you get | Functional and structural information on tumor associated genes |
| What you can do | Text search, search for oncogenic domains by protein sequence input, perform consensus analysis on two or more results of a search, browse by chromosome. |
| Literature & tutorials | Bioinformatics 2013 29: 420-427.  Tutorial available from home page of site |
| Updated | Last modified 10/03/2014 (not current) |
|  | |
| Tumor Gene Family Databases (TGDBs) | |
| URL | <http://www.tumor-gene.org/tgdf.html> |
| What you get | Information on tumor genes collected from OMIM, GeneCards and literature. |
| What you can do | Search by gene names or symbols or by locus. Search across the entire tumor gene database, or search the breast cancer or oral cancer specific databases. |
| Literature & tutorials | No links to help or tutorials on home page  No abstract summary paper in Nucleic Acids Research Database Issue, but it is NAR Molecular Biology Database Collection entry number 155 |
| Updated | Last modified 11/25/2007 (not current) |
|  | |
| UCSC Cancer Genome Browser | |
| URL | <https://genome-cancer.ucsc.edu/> |
| What you get | Integrated cancer genomics and clinical data in a heat map-style browser view. Site also includes the deployable Xena browser, which supports analysis of user data with Galaxy for visualization in Xena. |
| What you can do | Select from available data to display, upload experimental data, download data, view and sort data by chromosome, gene or clinical features, display Kaplan-Meier survival plots, upload custom signatures |
| Literature & tutorials | Nucl. Acids Res. (28 January 2015) 43 (D1): D812-D817. doi: 10.1093/nar/gku1073  User guide and tutorial available on site |
| Updated | Continuously updated |
|  | |
| UMD BRCA1/BRCA2 databases (Now BRCA Share) | |
| URL | <http://www.umd.be/BRCA2/> and <http://www.umd.be/BRCA1/> |
| What you get | Genomic variants on the BRCA1 and BRCA2 genes |
| What you can do | Users must register for access. Government, non-profit and academic users can register for free. You must register for EACH (BRCA1 and BRCA2) database separately. Once registered, browse using the “Variants Classification” link, which shows all variants with validated classification in the last 6 months. Use the “Mutations” link on the left to view and access all mutations by phenotype, significance or location. Results display the location of the mutation, protein change, functional domain (if any), biological significance, validation, date and number of records supporting the mutation. Users can also perform insertion and deletion analyses. |
| Literature & tutorials | Nucl. Acids Res. (2012) 40 (D1): D992-D1002. doi: 10.1093/nar/gkr1160  No links to help or tutorials on home page |
| Updated | Since BRCA Share is a data sharing platform, data are shared and updated by users continuously. |
